# Effect of vitreomacular adhesion on the treatment outcomes in the STOP-Uveitis clinical trial for non-infectious uveitis

**DOI:** 10.1186/s12348-019-0179-6

**Published:** 2019-07-19

**Authors:** Muhammad Hassan, Nam V. Nguyen, Muhammad Sohail Halim, Rubbia Afridi, Mohammad Ali Sadiq, Samendra Karkhur, Erin Vigil, Selen Karabekirogullari, Quan Dong Nguyen, Diana V. Do, Yasir J. Sepah, for the STOP-Uveitis Investigators

**Affiliations:** 1 Byers Eye Institute, Spencer Center for Vision Research, Stanford University, 2370 Watson Court, Suite 200, Palo Alto, CA 94303 USA; 2 Ocular Imaging Research and Reading Center, Menlo Park, CA USA

**Keywords:** Vitreomacular adhesion, Non-infectious uveitis, Tocilizumab, STOP-Uveitis

## Abstract

**Purpose:**

To evaluate the role of vitreomacular adhesion (VMA) in visual and anatomic outcomes in patients with non-infectious uveitis.

**Design:**

Phase 2 clinical trial

**Participants:**

Data from the Safety, Tolerability, and Efficacy of Tocilizumab in Patients with Non-infectious Uveitis (STOP-Uveitis) study was analyzed.

**Methods:**

In the STOP-Uveitis study, patients with non-infectious uveitis (NIU) received monthly intravenous infusions of either 4 or 8 mg/kg tocilizumab until month 6 (M6). Spectral domain optical coherence tomography (SD-OCT) images of patients that completed M6 of the study were analyzed at baseline to stratify the patients by the presence (VMA+) or absence (VMA−) of VMA. Patients with vitreomacular traction (VMT) or epiretinal membrane causing structural abnormalities within center 1 mm were excluded. All images were graded by two independent graders.

**Main outcome measures:**

Mean change in best-corrected visual acuity (BCVA), central retinal thickness (CRT), and vitreous haze (VH) at M6.

**Results:**

Out of 37 patients randomized in the STOP-Uveitis study, 48 eyes (27 patients) were eligible based on the study criteria. At baseline, 19 eyes were classified as VMA+, and 32 eyes were classified as VMA−. The distribution of two doses of TCZ (4 mg/kg and 8 mg/kg) were similar between the two groups. At M6, the mean improvement in BCVA was 2.00 ± 5.3 and 6.50 ± 7.98 letters in the VMA+ and VMA− groups, respectively (*p* = 0.02). The mean improvement in CRT was 34.85 ± 72.36 and 80.37 ± 157.21 μm in the VMA+ and VMA− groups, respectively (*p* = 0.18). Similarly, the mean change in VH was − 0.65 ± 0.47 and − 0.76 ± 0.71 in the VMA+ and VMA− groups, respectively (*p* = 0.32). Out of 16 eyes with VMA at baseline, 3 eyes developed posterior vitreous detachment (PVD) at M6. The mean change in BCVA was significantly higher (*p* = 0.02), while CRT and VH score were similar (*p* > 0.05) in eyes with PVD compared to eyes with persistent VMA.

**Conclusions:**

The absence of VMA or development of PVD in eyes with VMA seems to have a beneficial effect on the vision of subjects receiving treatment for uveitis. Therefore, patients with uveitis should be assessed using SD-OCT for the presence of vitreomacular interface abnormalities.

## Introduction

Uveitis is characterized by ocular inflammation that along with its complications accounts for 5–20% cases of preventable blindness in the developed world and up to 25% of cases in the developing countries [1]. Due to its heterogeneity, management of uveitis poses a great challenge for clinicians. The main goal in the management of uveitis is to control the inflammation and prevent recurrences.

Active research directed towards understanding the underlying pathophysiology of uveitis has led to development of a wide variety of drugs targeting several pathways believed to be responsible for the disease [2]. Although a number of these agents have shown efficacy in controlling inflammation in various clinical studies ranging from steroids to novel steroid-sparing agents, approximately 50% of the patients in these studies still are unable to demonstrate visual gains of 10 letters or more [2]. Such variation in response can be attributed to multiple causes such as duration and severity of the disease as well as environmental and genetic factors [3–5].

Vitreomacular interface (VMI) diseases are a spectrum of disorders characterized by aberrant attachment of the vitreous to the surface of the retina leading to pathologic manifestations [6]. The effects of VMI disorders on treatment outcomes have been explored in a variety of diseases such as age-related macular degeneration (AMD) and diabetic macular edema (DME) [7–12]. These abnormalities include vitreomacular traction, epiretinal membrane, and vitreomacular adhesion (VMA). In a retrospective study, the role of VMI abnormalities has been studied in patients with uveitic macular edema receiving intravitreal therapy [13]. However, the role of VMA in patients with non-infectious uveitis receiving systemic immunosuppressive therapy has not been evaluated previously. Therefore, in this analysis, we assessed the prognostic value of the presence or absence of VMA on treatment outcomes in patients with non-infectious uveitis receiving systemic therapy.

## Methods

Data from the Safety, Tolerability, and Efficacy of Tocilizumab in Patients with Non-infectious Uveitis (STOP-Uveitis) study was utilized for this study [14]. The STOP-Uveitis study was a multicenter, randomized, open-label clinical trial designed to assess the safety and efficacy of repeated intravenous (IV) infusions of 2 doses of TCZ (4 mg/kg and 8 mg/kg) in subjects with non-infectious uveitis. Starting at baseline, study participants in both study groups received monthly TCZ until the primary endpoint (month 6).

The STOP-Uveitis is registered at www.clinicaltrials.gov under the identifier NCT01717170 and was conducted in compliance with the US Code of Federal Regulations Title 21, the Declaration of Helsinki, and the Harmonized Tripartite Guidelines for Good Clinical Practice (1996). The study was approved by local institutional review boards for selected sites and by a central review board for others. Signed informed consent was obtained from all the participants of the study.

In this sub-study, the two study groups from the STOP-Uveitis clinical trials were combined and classified into two groups based on the presence (VMA+) or absence (VMA−) of VMA. Data from the study and fellow eyes which had confirmed the diagnosis of uveitis and fulfilled the inclusion and exclusion criteria were analyzed. Each eye was treated as an individual case in this study.

### Inclusion and exclusion criteria

Patients were included in the study if they met the following criteria: (1) participation in the STOP-Uveitis clinical trial and completion of the month 6 visit, and (2) availability of spectral domain optical coherence tomography (SD-OCT) images (Spectralis; Heidelberg Engineering, Heidelberg, Germany) of gradable quality. Patients with any degree of VMT, as defined in the published literature, and epiretinal retinal membrane causing significant tractional changes in the central 1 mm of the fovea were excluded from the study analysis [6, 15]. Inclusion and exclusion criteria of the STOP-Uveitis study have been published previously [14].

### Vitreomacular adhesion detection on spectral domain optical coherence tomography

The VMA status of both study and fellow eyes of subjects was assessed using SD-OCT images from the eligible subjects at the baseline and month 6 visit by two independent graders (NN and MH); a third senior grader was employed in cases of disagreements. The subjects were classified into either VMA+ or VMA− groups. The International Vitreomacular Traction Study group (IVTS) classification was used to define the presence of VMA [6]. The IVTS defines VMA as the presence of detachment of peri-foveal vitreous cortex from the retinal surface along with attachment of vitreous cortex within 3-mm radius of the fovea and no secondary changes in the foveal contour or underlying retinal tissue. The VMA is further classified by the size of the adhesion area into focal (< 1500 μm) or broad (≥ 1500 μm).

### Outcome measures

Mean change in best-corrected visual acuity (BCVA), defined as the numbers of Early Treatment Diabetic Retinopathy Study (ETDRS) letters read at 4 m, from baseline to month 6 was assessed in the two study groups. Mean change in CRT as measured by the SD-OCT from baseline to month 6 was also evaluated for the two study groups. Mean change in vitreous haze (VH) score was assessed utilizing the Standardization of Uveitis Nomenclature (SUN) scale from baseline to month 6.

### Statistical analysis

Stata V14.1 (Stata Corp, TX) was used for all statistical analysis. Frequencies were compared using the chi-square test. Wilcoxon’s signed-rank test was used to assess the differences in BCVA, CRT, and VH between baseline and month 6 of both study groups. The Mann-Whitney *U* test was utilized for assessment of mean differences in BCVA, CRT, and VH between the 2 groups at month 6.

## Results

A total of 37 patients were included in the STOP-Uveitis study. Out of 37 patients, 27 patients (48 eyes) were included in this sub-study analysis based on the inclusion and exclusion criteria. Twenty-six eyes were excluded from the study as they failed to meet the study criteria: 19 eyes did not have gradable SD-OCT images and 7 eyes had epiretinal membrane. At baseline, 16 eyes (33.33%) were classified into VMA+ group, and 32 eyes (66.66%) were classified into VMA− group. Baseline characteristics of the two groups are shown in Table 1. There were no statistical differences between the baseline characteristics of the two study groups (Table 1). The distribution of two doses of IV TCZ (4 and 8 mg/kg) was also similar between the two study groups.

**Table 1:** Baseline characteristics of the study population

Characteristics	Vitreomacular adhesion present (VMA+)	Vitreomacular adhesion absent (VMA−)	*p* value
Mean age ± SD (years)	41 ± 18.5	46 ± 19.0	0.46
Female gender (%)	40.00	65.63	0.38
Caucasian (%)	66.77	90.63	0.08
Mean BCVA at baseline (ETDRS letters)	60.68 ± 17.46	61.13 ± 16.46	0.93
Mean baseline CRT (μm)	319.06 ± 132.01	351.47 ± 174.97	0.48
Mean baseline VH	1.17 ± 0.98	1.19 ± 0.91	0.94

### Best-corrected visual acuity

**Fig. 1:**
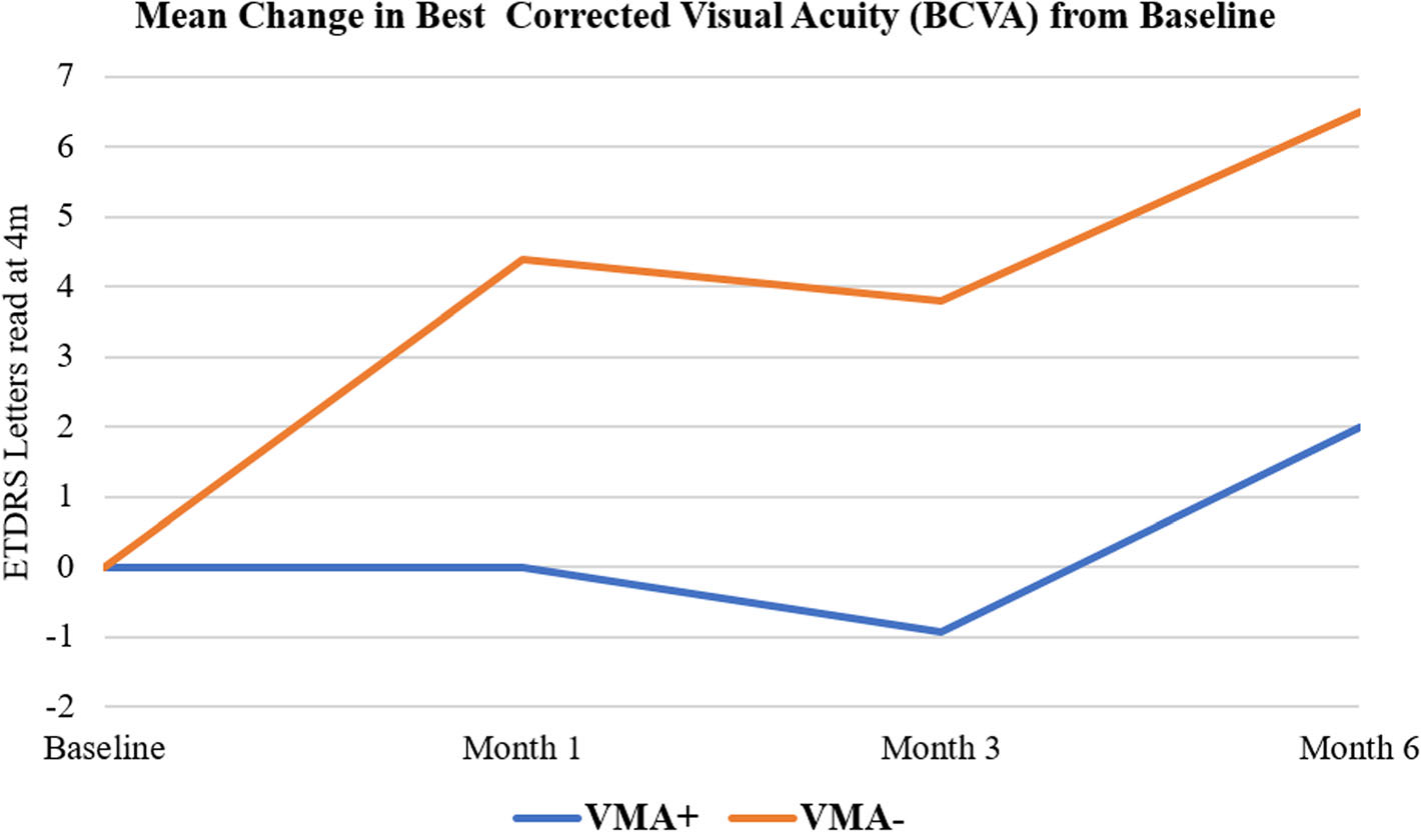
Mean change in best-corrected visual acuity. The mean change in best-corrected visual acuity (BCVA) from baseline at different study intervals for the 2 study groups. ETDRS, Early Treatment Diabetic Retinopathy Study; VMA+, vitreomacular adhesion present; VMA−, vitreomacular adhesion absent

At month 6, the mean change in BCVA in VMA+ group was 2.00 ± 5.31 letters, whereas the mean change in BCVA in VMA− group was 6.50 ± 7.98 letters. The mean improvement in BCVA in VMA+ group was significant from the baseline (*p* < 0.05). However, the mean improvement in BCVA in VMA− group was not statistically significant from the baseline (*p* > 0.05). The difference in the mean change in BCVA between the two groups was statistically significant (*p* = 0.02). Figure 1 shows the mean change in BCVA from baseline to month 6 in the 2 study groups.

### Central retinal thickness

**Fig. 2:**
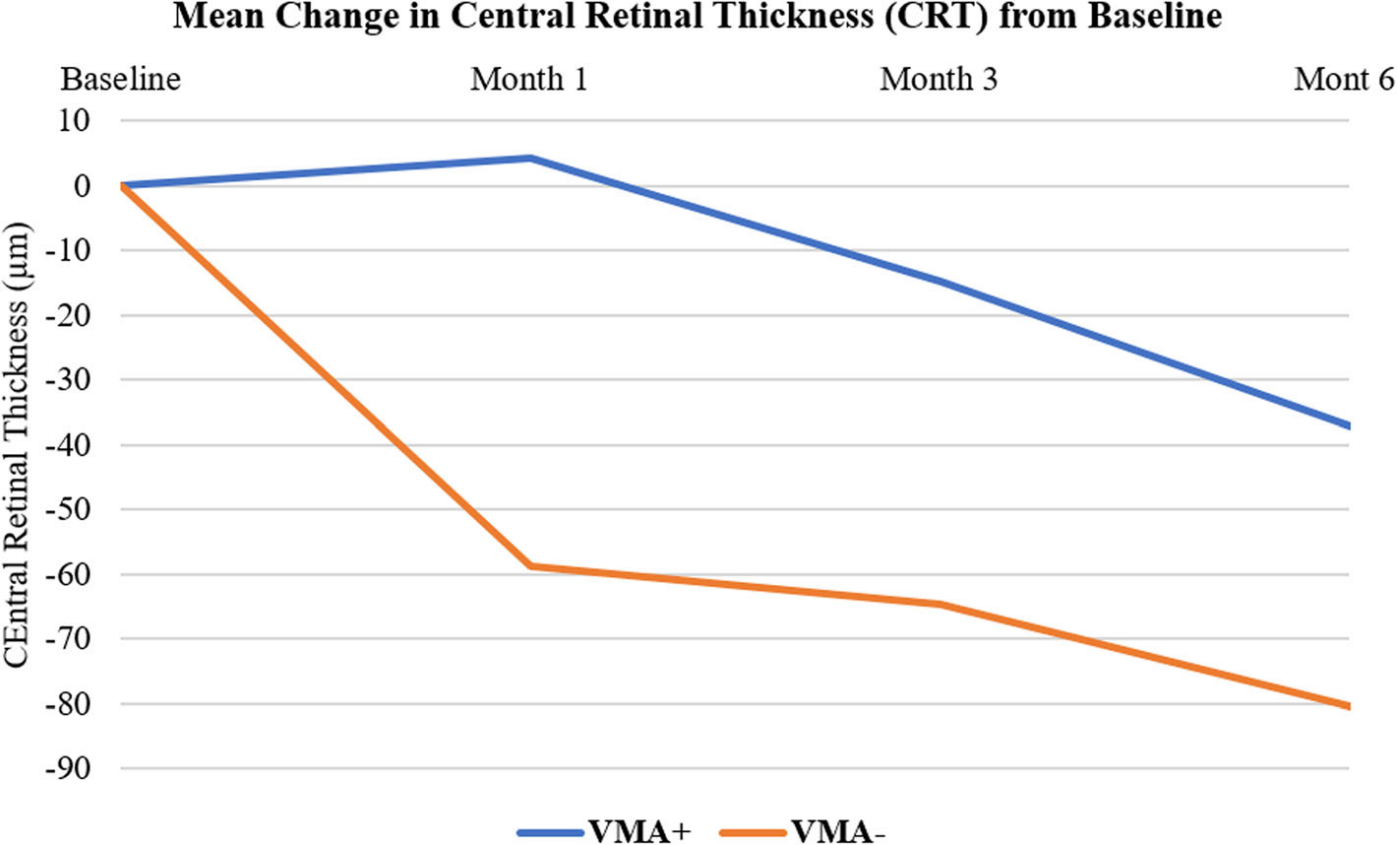
Mean change in central retinal thickness. The mean change in central retinal thickness (CRT) from baseline at different study intervals for the 2 study groups. VMA+, vitreomacular adhesion present; VMA−, vitreomacular adhesion absent

At month 6, the mean reduction in CRT was 34.85 ± 72.36 and 80.37 ± 157.21 μm in the VMA+ and VMA− groups, respectively. The mean reduction in the CRT at month 6 was significant compared to baseline in both VMA+ and VMA− groups (*p* < 0.05). The difference in the mean change in CRT between two groups was however not statistically significant (*p* = 0.18). Figure 2 illustrates changes in the CRT from baseline at different time points for the study groups.

### Vitreous haze

**Fig. 3:**
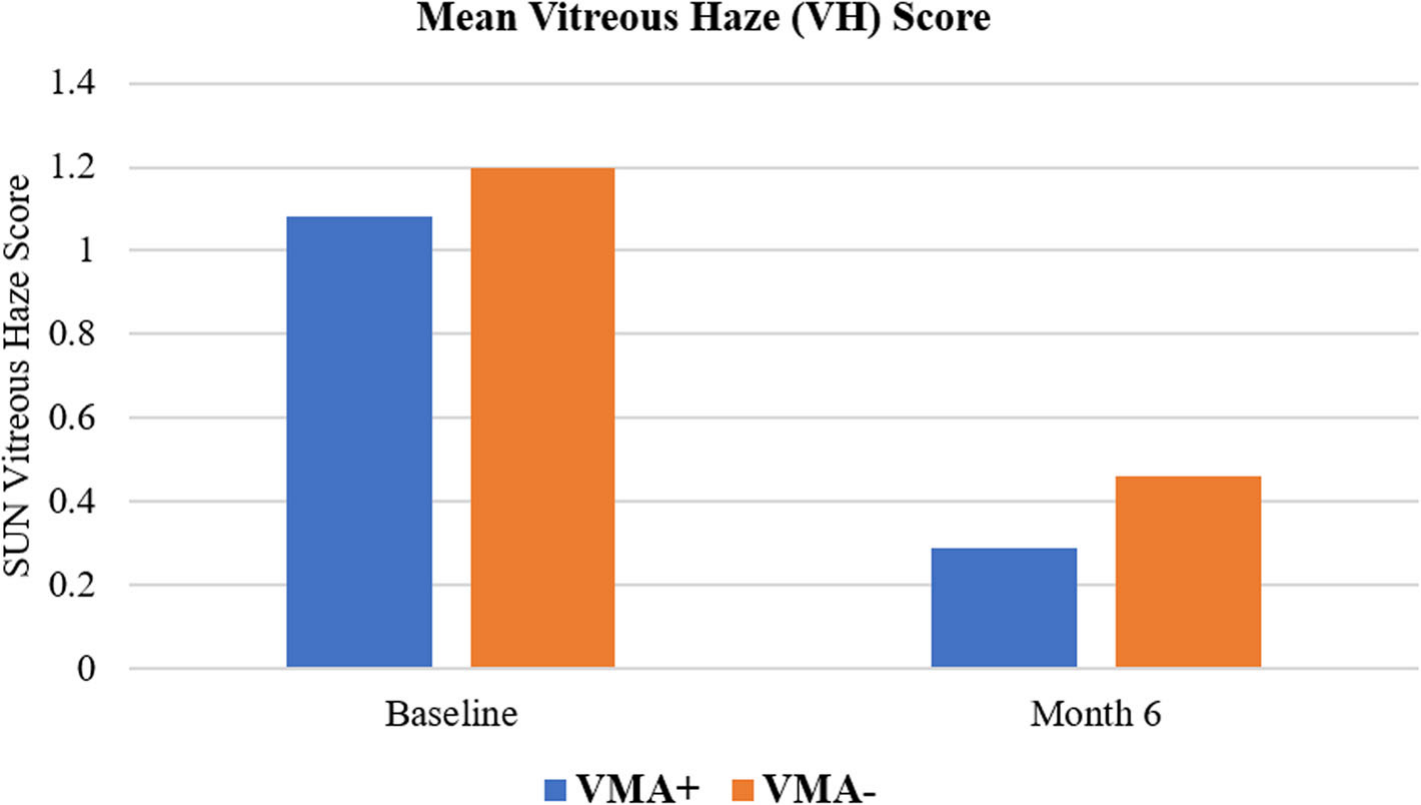
Mean vitreous haze score. The mean vitreous haze (VH) score at baseline and month 6 for the 2 study groups. VMA+, vitreomacular adhesion present; VMA−, vitreomacular adhesion absent

At month 6, the mean change in VH score in VMA+ group was − 0.65 ± 0.47, and in VMA− group was − 0.76 ± 0.71. Both the groups demonstrated significant improvement in the VH score from baseline (*p* < 0.05). However, the difference in VH score improvement between two groups was not statistically significant (*p* = 0.32). Figure 3 shows the mean VH score at baseline and month 6 for the 2 study groups.

### Vitreomacular interface status at month 6

Of the 16 VMA+ eyes at baseline, PVD occurred in 3 eyes at month 6, whereas 13 eyes had no change in the VMA status. The mean change in BCVA in eyes with PVD from baseline to month 6 was 8.00 ± 5.29 letters, which was significant compared to 0.62 ± 3.50 letters in eyes with persistent VMA at month 6 (*p* = 0.02). However, the mean reduction in the CRT in eyes which developed PVD (85.67 ± 109.10 μm) was not significantly different than the mean reduction in CRT in eyes with persistent VMA (25.81 ± 64.51 μm) (*p* > 0.05). Similarly, the mean change in the VH score was − 0.55 ± 0.50 and − 0.67 ± 0.58 in the eyes which developed PVD and eyes with persistent VMA, respectively, and it was not significantly different (*p* > 0.05).

### Focal versus broad vitreomacular adhesion

SD-OCT analysis of the eyes with VMA at baseline showed that 14 eyes had broad VMA while 2 eyes had focal VMA. Mean change in the BCVA was 1.43 ± 4.47 and 6.00 ± 11.31 letters in eyes with broad and focal VMA from baseline to month 6, respectively. Mean reduction in CRT was 26.89 ± 64.12 and 104.5 ± 150.61 μm in eyes with broad and focal VMA, respectively. Similarly, the mean change in VH score in eyes with broad VMA was − 0.55 ± 0.47 compared to − 1.00 ± 00 in eyes with focal VMA. There was no statistically significant difference between the eyes with broad and focal VMA in any of the parameters assessed (*p* > 0.05).

### Edema status

**Fig. 4:**
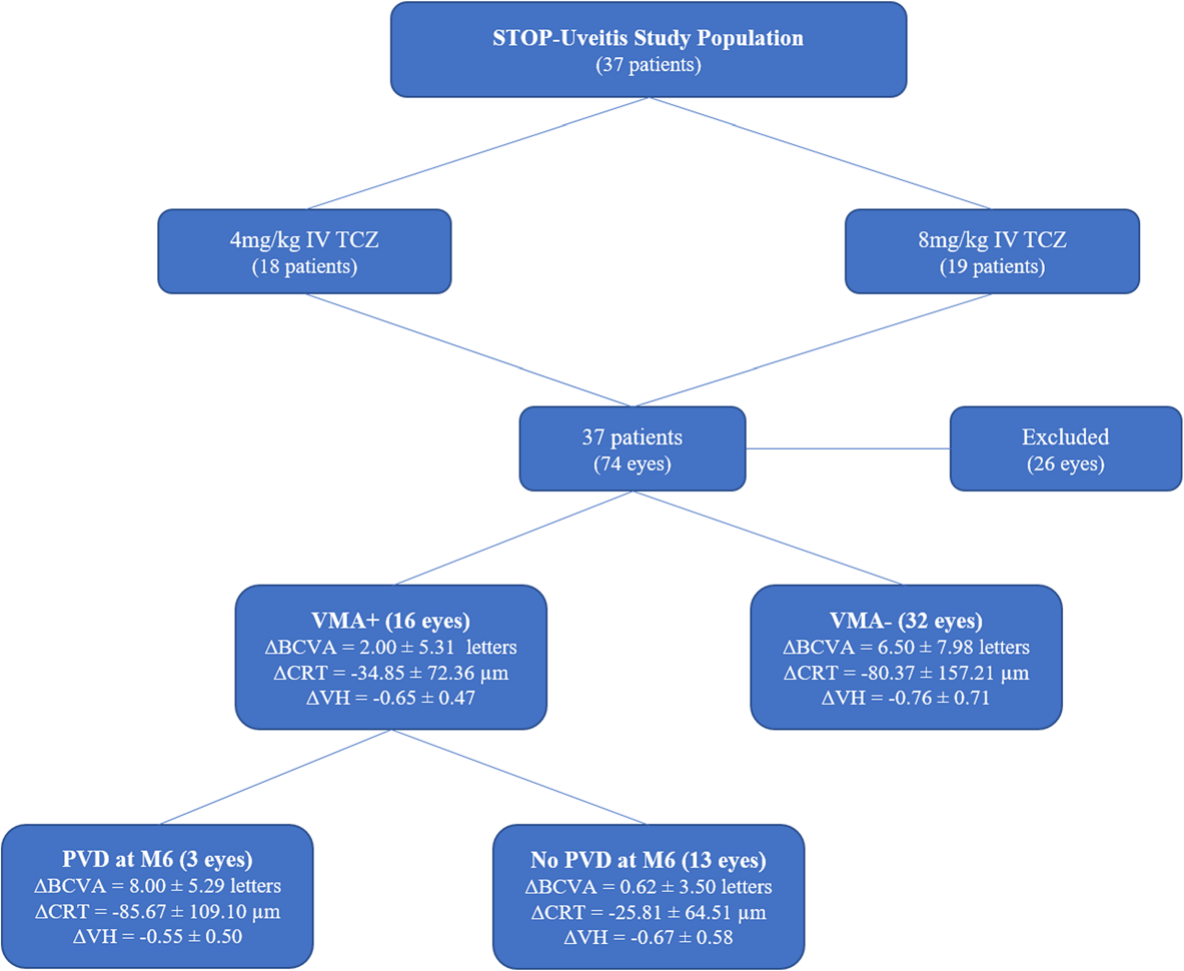
Study population. Flowchart outlining the characteristics of the study population. BCVA, best-corrected visual acuity; CRT, central retinal thickness; IV, intravenous; TCZ, tocilizumab; VH, vitreous haze; VMA+, vitreomacular adhesion present; VMA−, vitreomacular adhesion absent; Δ, mean change from baseline to month 6

At baseline, 12 eyes had presence on macular edema on the SD-OCT images (3 in VMA+ and 9 in VMA− group). The mean improvement in BCVA at month 6 in eyes with edema at the baseline was 3.00 ± 11.53 and 12.44 ± 10.68 in the VMA+ and VMA− groups, respectively (*p* > 0.05). The mean reduction in CRT at month 6 in eyes with edema at baseline in the VMA+ group was 167.33 ± 96.24 compared to 243.50 ± 217.98 in the VMA− group (*p* > 0.05). Similarly, the mean change in the VH score at month 6 in eyes with edema at baseline was − 0.25 ± 1.07 and − 1.00 ± 1.00 in the VMA+ and VMA− groups, respectively (*p* > 0.05). Figure 4 summarizes the important characteristics of the populations which were analyzed.

## Discussion

VMI abnormalities are a spectrum of disorders characterized by aberrant attachment of the vitreous to the surface of the retina leading to pathologic manifestations. Some of these abnormalities like vitreomacular tractions (VMT) and macular holes can overtly cause visual and anatomic abnormalities of the eye and are therefore generally excluded from the clinical studies looking at the efficacy of new therapies. On the other hand, abnormalities like VMA and PVD may present as clinically asymptomatic conditions which can only be detected by the OCT. However, recent studies have shown that the presence of VMA and PVD may affect the visual and anatomic outcome in subjects receiving anti-vascular endothelial growth factor (anti-VEGF) agents for AMD and DME [7–12]. Similarly, Munk et al. also demonstrated the effects of VMI configurations on patients receiving intravitreal (IVT) therapy for uveitic macular edema that was treated with intravitreal therapy [13]. However, the effects of VMA on the visual and anatomic outcomes of subjects with uveitis receiving systemic immunosuppressive therapy have not been explored previously.

The results of the index study showed that the eyes of the patients with uveitis without VMA demonstrate significant improvement in the BCVA from baseline after treatment with IV TCZ. This improvement in the BCVA in the VMA− eyes was significantly greater than the VMA+ eyes, which even failed to demonstrate significant improvement in BCVA from the baseline, demonstrating the importance of vitreomacular interface in eyes with ocular inflammation with VMA contributing to the suboptimal visual acuity. Upon further analysis, the subjects who had VMA at baseline and developed PVD had significantly greater improvement in the BCVA compared to the eyes with persistent VMA. A number of previous studies evaluating the role of VMA in patients with AMD receiving IVT anti-vascular endothelial growth factor (anti-VEGF) agents have indicated poor visual outcome and need for more frequent treatment in eyes with the presence of VMA [8–11]. On the contrary, mixed results have been reported about the effect of VMA on BCVA in eyes with DME [7, 12]. Sadiq et al. showed better final vision in VMA+ eyes receiving anti-VEGF therapy for DME. However, on further evaluation, they attributed the finding to younger population in the VMA+ group and development of PVD in a significant number of subjects with VMA at baseline [7]. They demonstrated that patients who developed PVD by 6 months had better vision compared to those with persistent VMA which is similar to what we noted in our study. Development of PVD has been shown to be associated with better visual outcomes in AMD as well [11]. Similarly, on comparison of eyes with focal and broad VMA in our study, the BCVA seemed to be better in eyes with focal VMA which are likely to develop PVD compared to broad VMA.

Munk et al. evaluated the role of VMI configurations in subjects with uveitic macular edema (UME) and found that the presence of PVD was associated with a significant decrease in the CRT compared to baseline and other VMI configurations [13]. In our study, both VMA+ and VMA− eyes demonstrated a significant decrease in the CRT compared to baseline; however, the results between the two groups were not significant. On further exploration, the subset of eyes which developed PVD by month 6 may be responsible for a significant decrease in CRT from the baseline noted in the VMA+ group. The decrease in the CRT in eyes with PVD (85.67 ± 109.10) was similar to VMA− eyes (80.37 ± 157.21) and different than the VMA+ eyes with persistent VMA at month 6 (34.85 ± 72.36 μm). Similarly, eyes with focal VMA which are more likely to develop PVD had a higher decrease in CRT compared to eyes with broad VMA (104.5 ± 150.61 vs 26.89 ± 64.12, respectively), most likely because it is likely to be easier to develop a PVD if the area of adhesion is smaller. The difference between the groups in our study was not significant probably because unlike Munk et al., whose study was specifically for UME, our study population started off with a lower baseline CRT and our study was not powered to assess the effects of edema due to a smaller number of eyes with edema at baseline (12 out of 48 eyes) [7]. On additional analysis of the 12 eyes with the presence of edema at the baseline, the mean change in BCVA and CRT was higher in the VMA− group (12.44 ± 10.68 and 243.50 ± 217.98, respectively) compared to the VMA+ group (3.00 ± 11.53 and 167.33 ± 96.24, respectively). Even though this difference was insignificant due to the small sample size, the trend was similar to what we noted in the overall analysis of the study.

Interestingly, Munk et al. failed to demonstrate the effect of VMA and PVD on the BCVA of the study subjects despite a significant decrease in the CRT in the PVD group [9]. There could be various reasons for this discrepancy in the results between the two studies. They attributed the smaller increase in visual function to that fact that in eyes with CME, other factors like ellipsoid zone disruption, ELM disruption, and number of alive axons may also play a role in visual function after resolution of the edema [16, 17]. Therefore, our study had fewer number of eyes with edema/UME, and hence, the visual outcomes were potentially less influenced by these factors. Additionally, all the patients included by Munk et al. were retrospectively selected and had received only one treatment at baseline with either IVT triamcinolone, IVT bevacizumab, or a dexamethasone implant followed by a 3-month follow-up period. Therefore, longer treatment period with a single agent as in our study may be needed for significant change in visual acuity of the patients with uveitis.

Based on the results of our study, the presence or absence of VMA does not seem to have any significant effect on the VH score in eyes with uveitis. Both VMA+ and VMA− eyes demonstrated significant reduction in VH score from baseline; however, there was no difference between the two groups.

The exact pathogenesis of how VMI configurations can influence the visual and anatomic outcomes in various diseases are not known; however, several possible mechanisms have been suggested. It has been hypothesized that VMA is associated with chronic localized inflammatory state with confinement of inflammatory molecules in the premacular hyaloid along with prevention of diffusion of nutrients and oxygen to the macula [9, 18, 19]. Such localized inflammatory state may decrease the efficacy of systemic anti-inflammatory therapy penetrating the inflamed areas, resulting in poor visual and anatomic outcome. Additionally, PVD has been associated with increased convention fluxes and diffusion of nutrients and oxygen along with clearance of inflammatory cytokines from the area [18, 20, 21]. Therefore, detachment of posterior hyaloid in eyes with VMA may clear this reservoir of the local inflammatory cells and cytokines and result in better outcomes.

The strengths of our analysis included outcomes from a well-characterized prospective study design of a multicentered clinical trial (the STOP-Uveitis study) with a mandatory treatment regimen for 6 months with a single drug. The SD-OCT scans were captured using a standardized protocol, and assessments of VMI configurations were performed in a formal reading center environment by 2 masked graders. We utilized a validated system for the classification of VMI configurations. To the best of our knowledge, our analysis is the first study to evaluate the role of VMA in determining visual and anatomic outcomes on eyes with non-infectious uveitis receiving a systemic anti-inflammatory agent, eliminating the potential confounding effects of intravitreal injections on VMI and VMA.

Possible limitations of our study include a relatively small sample size especially for subgroup analyses where our results showed a trend but failed to achieve statistical significance. Additionally, our analysis was an additional, post hoc exploratory analysis of the STOP-Uveitis study, which was primarily designed to assess the role of two different doses of IV TCZ on outcomes in patients with non-infectious uveitis.

In conclusion, it is important to identify the structural characteristics which influence the treatment outcomes in patients with uveitis. The results of our study suggest that patients with uveitis without VMA or who have VMA but subsequently develop PVD may demonstrate better visual gains after treatment with systemic immunosuppressive agents such as TCZ. Therefore, patients with uveitis should be assessed using SD-OCT for the presence of VMI abnormalities at the initiation of therapy and periodically thereafter.

## Data Availability

The datasets used during the current study are available from the corresponding author on reasonable request.

## Abbreviations

BCVA: Best-corrected visual acuity
CRT: Central retinal thickness
IVT: Intravitreal
IVTS: International Vitreomacular Traction Study group
PVD: Posterior vitreous detachment
SD-OCT: Spectral domain optical coherence tomography
SUN: Standardization of Uveitis Nomenclature
TCZ: Tocilizumab
VEGF: Vascular endothelial growth factor
VMA: Vitreomacular adhesion
VMA–: Vitreomacular adhesion absent
VMA+: Vitreomacular adhesion present
